# SHEDs and BMSCs exhibit distinct lineage preferences in HUVECs dynamic spheroid co‐cultures: vascular versus osteogenic commitment

**DOI:** 10.1002/btm2.70091

**Published:** 2025-11-17

**Authors:** Soukaina El Hajj, Caroline Gorin, Martial Bankoué Ntaté, Romane Lesieur, Elina Casas, Catherine Chaussain, Didier Letourneur, Joelle Amédée, Hervé Duval, Bruno Paiva Dos Santos, Bertrand David

**Affiliations:** ^1^ Université Paris‐Saclay, CentraleSupélec, ENS Paris‐Saclay, CNRS LMPS ‐ Laboratoire de Mécanique Paris‐Saclay Gif‐sur‐Yvette France; ^2^ Université Paris Cité, Université Sorbonne Paris Nord, Dental School, INSERM UMR‐S 1333 Laboratory for Oral Health (LOH) Montrouge France; ^3^ Assistance Publique des Hôpitaux de Paris (AP‐HP) Service de médecine bucco‐dentaire, GH Nord Paris France; ^4^ Université Paris‐Saclay, CentraleSupélec LGPM ‐ Laboratoire de Génie des Procédés et Matériaux Gif‐sur‐Yvette France; ^5^ Université de Bordeaux, INSERM U1026 Laboratoire de Bioingénierie Tissulaire (BioTis) Bordeaux France; ^6^ Université Paris Cité, Université Sorbonne Paris Nord, INSERM U1148 Laboratoire de Recherche Vasculaire Translationnelle (LVTS), Hôpital Bichat Paris France

**Keywords:** 3D cell culture, dental stem cells, perfusion bioreactor, porous hydrogel, spatial reorganization, spheroids

## Abstract

Stem cells from human exfoliated deciduous teeth (SHEDs) offer a promising alternative to bone marrow‐derived mesenchymal stem cells (BMSCs) for bone tissue engineering due to their accessibility, high proliferative potential, and multipotency. In this study, we compared the osteogenic and angiogenic potential of two mesenchymal stem cells subpopulations, SHEDs and BMSCs, when co‐cultured with human umbilical vein endothelial cells (HUVECs) into spheroids over a period of 28 days in porous pullulan/dextran scaffolds loaded with hydroxyapatite (HAp) particles as the sole osteoinductive cue. Spheroids were cultured under static and dynamic conditions, with the latter employing a perfusion flow bioreactor to enhance solute transport and oxygenation. Dynamic culture conditions significantly improved cell viability compared to static culture (85% vs. 54% at Day 28), maintained spheroid integrity, and promoted the expression of angiogenic markers, such as the cluster of differentiation 31 (CD31) and von Willebrand factor (vWF), which under static culture were largely confined to the spheroid periphery. Furthermore, alpha‐smooth muscle actin/neural‐glial‐antigen 2 (αSMA/NG2) and CD31/NG2 colocalization reflected close spatial associations between SHEDs and HUVECs, suggesting a supportive perivascular interaction under dynamic culture. In the presence of HUVECs, we found that HAp particles alone were insufficient to induce robust osteogenic differentiation in SHEDs. Weak alkaline phosphatase activity, minimal osteopontin and osteocalcin expression, and incomplete mineralization were observed under both static and dynamic conditions. In contrast, BMSC/HUVEC spheroids exhibited robust osteogenic differentiation and consistent mineral deposition. These results show intrinsic differences in the behavior of SHEDs and BMSCs when co‐cultured with endothelial cells; while BMSCs tend to favor osteogenesis, SHEDs appear to adopt a more perivascular or pericytic behavior.

**Translational Impact Statement**

Cell‐based strategies for bone regeneration hold strong promise, particularly for high‐risk patient populations with impaired healing. Preclinical evidence supports the use of mesenchymal stem cell/endothelial co‐cultures to enhance both osteogenesis and vascularization. Our study compares stem cells from human exfoliated deciduous teeth (SHEDs) and bone marrow‐derived mesenchymal stem cells (BMSCs) co‐cultured with endothelial cells under dynamic 3D culture and revealed distinct lineage trajectories shaped by spatial organization. At the biological level, BMSCs primarily supported bone formation, whereas SHEDs promoted vascular network development. These findings provide guidance for rational cell source selection to optimize the functionality of engineered constructs. At the systems level, combining perfusion with SHED‐based constructs may represent a promising prevascularization strategy to improve implant integration.

## 1 INTRODUCTION

Since its emergence in the 1980s, stem cell research has enabled the promise of personalized and regenerative medicine and revolutionized our understanding of diseases by improving physiological modeling and biomimicry. Nevertheless, decades later, stem cell research still faces significant challenges, beginning with the limited availability of cell sources, as their extraction often involves invasive procedures or access to rare donor tissues. Culturing stem cells is an expensive and tedious process, demanding specialized equipment, precise media and culture conditions, and skilled expertise to preserve their viability and successfully direct their differentiation pathways.^1^, ^2^ In the context of bone tissue engineering, bone marrow‐derived mesenchymal stem cells (BMSCs) have been considered the gold standard of osteogenic precursors due to their robust ability to differentiate into osteoblasts.^3^, ^4^, ^5^ However, BMSCs are challenging to extract and culture due to their low abundance in tissues, requiring laborious and invasive procedures like bone marrow aspiration to isolate them.^6^ Once extracted, BMSCs have a limited lifespan in vitro with reduced proliferation and differentiation potential after several passages, depending on the donor's health and age, yielding small amounts of cells in vitro and complicating large‐scale experiments in common research facilities.^7^, ^8^

In recent years, mesenchymal stem cells (MSCs) from stem cells from human exfoliated deciduous teeth (SHEDs) have emerged as promising candidates in regenerative medicine and tissue engineering applications. The use of SHEDs presents a significant advantage in developing therapeutic protocols due to their accessibility and their potential to differentiate into various cell types, including osteoblasts, chondrocytes, adipocytes, hepatocytes, and even neural cells.^9^, ^10^ This MSC‐subpopulation, located in the dental pulp of naturally exfoliated primary teeth, is easy to harvest and expand.^11^, ^12^ This offers practical and non‐invasive isolation methods that pose minimal ethical concerns to traditional MSC‐extraction methods that inflict additional surgeries which may increase the risk of postoperative morbidities and patient discomfort.^13^ SHEDs have been shown to share many characteristics with BMSCs, including their multipotency and capacity for self‐renewal.^10^ For instance, various mesenchymal markers were found to be positively and strongly expressed such as stromal cell surface anigen‐1 (STRO‐1), CD44, CD90, CD73, and CD105, confirming their mesenchymal profile, which explains their ability to differentiate into mesodermal lineages.^10^, ^14^, ^15^ However, unlike other mesenchymal populations, SHEDs possess the unique and remarkable potential to differentiate into ectodermal lineages, including neural cells, due to their neural crest origin.^16^ This characteristic makes them an attractive choice for researchers aiming to develop complex tissue models, particularly in spheroid and organoid research, where 3D communication and interaction between different cell types is of essence. Moreover, SHEDs' superior proliferative capacity and ease of ex vivo expansion make them cost‐effective mesenchymal candidates and more readily manipulated in vivo.^17^, ^18^, ^19^, ^20^

Among dental stem cells, several studies have shown that SHEDs surpass the dental pulp stem cells harvested from permanent teeth (DPSCs) in osteogenic differentiation, despite DPSCs being more extensively studied in regenerative medicine research. SHEDs exhibit stronger mineralization and higher expression levels of osteogenic markers such as alkaline phosphatase (ALP) and collagen type I (Col1), particularly at early passage numbers.^21^, ^22^ Unlike DPSCs, SHEDs have also been shown to form bone‐like structures in vivo, showing a reliable capacity for mineralized tissue formation.^23^ DPSCs demonstrate significant odontogenic differentiation and are more suited for dentin matrix formation,^24^ while SHEDs seem overall better adapted for applications requiring robust bone formation. Even when compared to more established MSC subpopulations in bone research, SHEDs displayed comparable osteogenic abilities to BMSCs on some occasions and superior osteogenic capabilities to adipose‐derived MSCs (AD‐MSCs).^25^, ^26^, ^27^

To develop prevascularized tissue constructs, the co‐culture of SHEDs or DPSCs with endothelial cells (ECs) creates a symbiotic environment with significant angiogenic potential.^28^, ^29^ In particular, SHEDs have demonstrated the ability to enhance microvascular network formation by secreting key pro‐angiogenic factors such as vascular endothelial growth factor (VEGF) and hepatocyte growth factor (HGF), which promote endothelial recruitment and support the deposition of collagen type IV (Col IV) in the vascular basement membrane.^28^, ^29^, ^30^ The ability of SHED/EC and DPSC/EC co‐cultures to improve vascularization has been validated throughout various experimental setups. When compared to solo SHED culture, SHED/EC co‐culture within polylactic acid (PLLA) scaffolds increased vessel density and formation.^31^ Transplantation of SHEDs or human umbilical vein endothelial cells (HUVECs) alone into immunodeficient mice failed to form functional vessels, but their co‐transplantation resulted in extensive vessels. SHEDs exhibited higher expression of VEGF, stromal cell‐derived factor‐1 alpha (SDF‐1α), and platelet‐derived growth factor receptor beta (PDGFRβ), while HUVECs expressed more VEGF receptors, chemokine receptor type 4 (CXCR4), and platelet‐derived growth factor subunit B (PDGF‐BB).^32^ Moreover, the interplay between DPSCs and ECs extends beyond angiogenesis and prevascularization methods to create an environment that is nurturing to odontogenesis and osteogenesis as well. SHEDs cultured with HUVECs formed lumenized capillaries in three‐dimensional (3D) hydrogels while maintaining their odontogenic potential near dentin surfaces.^28^ Similarly, DPSC/HUVEC spheroids enhanced vascular network formation while simultaneously promoting osteogenesis and odontogenesis through direct communication and paracrine signaling, especially when compared to DPSCs monoculture.^33^ In addition to direct cell–cell communication, the paracrine effects of DPSCs are central to their pro‐angiogenic potential. These cells secrete factors such as VEGF, HGF, monocyte chemoattractant protein‐1 (MCP‐1), and SDF‐1, which stimulate endothelial migration, tubulogenesis, and capillary‐like structure formation by activating the phosphoinositide 3‐kinase/protein kinase B (AKT) and the mitogen‐activated protein kinase/extracellular signal‐regulated kinase signaling pathways.^30^, ^34^, ^35^, ^36^

While co‐culture systems present promising potential for vascularized bone regeneration, the next step toward clinical translation lies in embedding these systems within scaffolds that both sustain long‐term viability and mimic physiological osteoinductive signals. Conventional in vitro osteogenic assays typically rely on pharmacological supplements such as dexamethasone, β‐glycerophosphate, and ascorbic acid to trigger differentiation, but these conditions poorly reflect the physiological context and present limited translational potential. In vivo, early osteoinduction arises primarily from the mineralized extracellular matrix and the release of calcium and phosphate ions within the healing microenvironment.^37^ Hydroxyapatite (HAp), the major mineral constituent of bone, has been shown to promote spontaneous osteogenic commitment of MSCs even in the absence of exogenous induction factors.^38^ Guided by this rationale, we established a simplified and biomimetic culture model in which HAp particles served as the sole osteoinductive cue, thereby enabling us to assess the intrinsic osteogenic and angiogenic properties of different stem cell populations under conditions closer to early in vivo bone healing. Among the candidate materials, pullulan/dextran/sodium (PUDNA) hydrogels have shown promising osteoinductive and osteoconductive properties both in vitro and in vivo, particularly when supplemented with HAp.^39^, ^40^ Building on the work of Bidarra et al. on angiogenic‐osteogenic synergy, co‐culturing BMSCs and HUVECs in spheroidal form within PUDNA scaffolds enhanced spatial organization and significantly improved osteogenesis compared to BMSC‐monocultures in critically sized femoral defects.^41^, ^42^ To overcome viability limitations inherent to long‐term spheroid culture while also recapitulating some biomechanical properties of the early bone defect microenvironment, a dynamic perfusion bioreactor was integrated into the culture system, resulting in improved viability, osteogenesis, and angiogenesis in immortalized BMSC/HUVEC spheroids compared to static conditions.^43^

In this context, the present study aims to advance the clinical translation of this tissue engineering strategy by replacing BMSCs with SHEDs, a highly accessible and scalable mesenchymal source with demonstrated pro‐osteogenic and pro‐angiogenic properties. Specifically, we investigated the osteogenic and angiogenic potential of SHED/HUVEC spheroids dynamically cultured within PUDNA/HAp composite scaffolds, without the addition of exogenous osteogenic factors. The objectives are twofold: (1) to compare the osteogenic performance of SHEDs to that of BMSCs under dynamic 3D culture conditions; and (2) to examine the spatial localization of SHEDs in relation to HUVECs, in order to understand their contribution to early endothelial network formation and osteogenesis.

## 2 MATERIALS AND METHODS

### 2.1 Preparation of porous pullulan/dextran scaffolds

**FIGURE 1:**
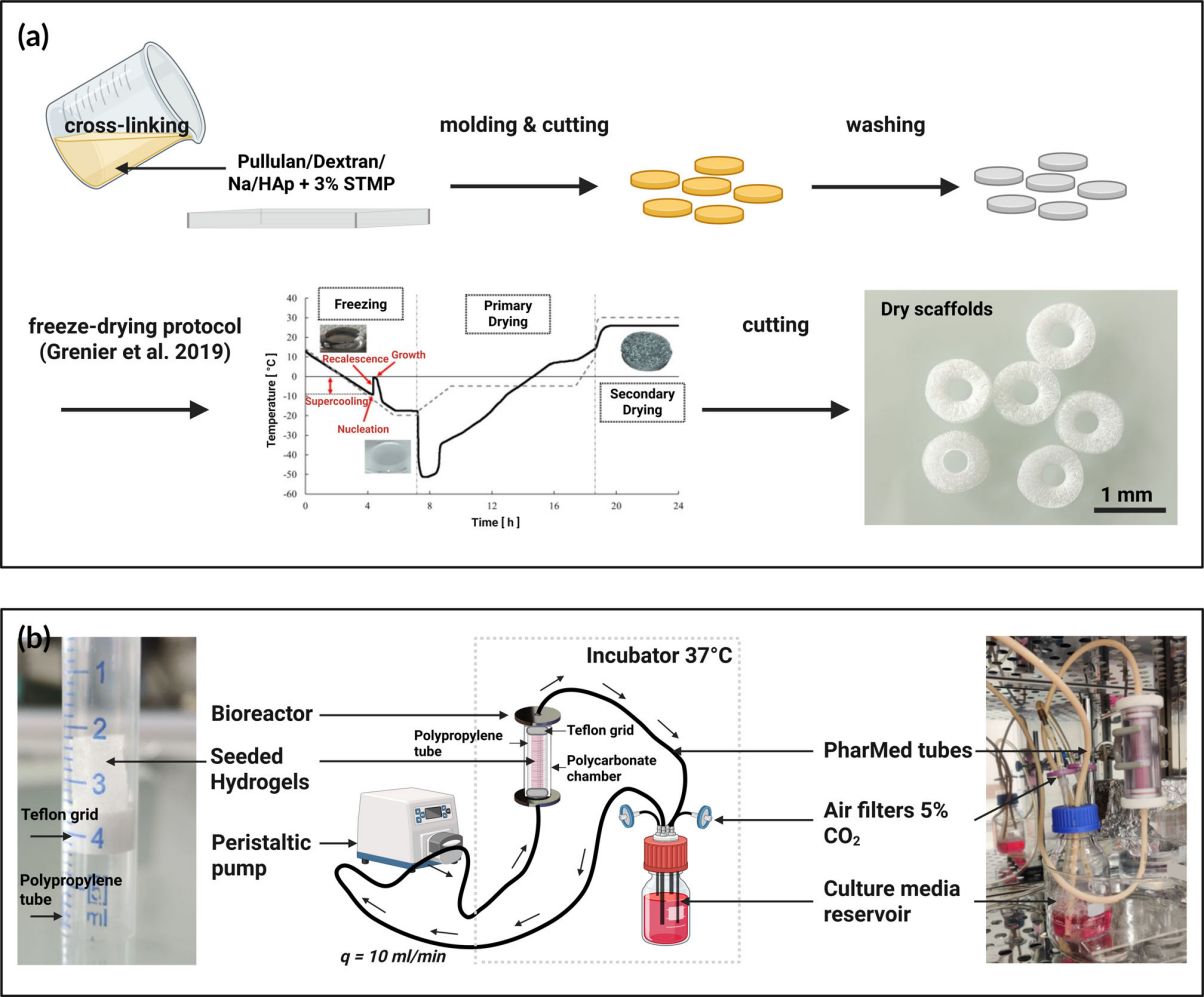
(a) Fabrication method of pullulan/dextran/hydroxyapatite (HAp) scaffolds involving crosslinking and freeze‐drying protocols as described by Grenier et al.^44^ Scale bar = 1 mm. (b) Dynamic culture setup: Cell‐laden scaffolds are centrally aligned and stacked inside a polypropylene tube and held in place between two teflon grids. The tube is then placed inside a polycarbonate chamber connected to a peristaltic pump and a culture media reservoir through silicone tubes, creating a perfusion circuit at 37°C, 5% CO_2_.

Porous scaffolds were prepared using a blend of pullulan (130 g L^−1^, Pullulan 200,000, Hayashibara, Japan) and dextran (43.3 g L^−1^, Dextran 500 kDa, Pharmacosmos), as previously described.^44^, ^45^, ^46^, ^47^ Crosslinking was achieved with sodium trimetaphosphate (STMP) (9.35 × 10^−2^ mol L^−1^) under saline (NaCl, 3.46 mol L^−1^) and alkaline conditions (NaOH, 0.953 mol L^−1^). HAp was incorporated by mixing 22.97 mL of HAp solution (36 mg L^−1^) and 17 mL of distilled water within the pullulan/dextran/sodium (PUDNA) mixture. After crosslinking, the gel solution was cast into a 1 mm‐thick sheet and baked for 20 min at 50°C to increase viscosity and harden the structure. The sheets were then cut into round discs (5.5 mm diameter) using a custom‐made stainless‐steel punch tool. The samples were neutralized using phosphate buffer (PBS 10,007×) and distilled water, then washed repeatedly in a saline solution (NaCl, 0.250 g L^−1^). The swollen hydrogel discs were placed horizontally on petri dishes in sets of 50 before freeze‐drying using a freeze‐dryer (MUT 004, Cryotech®, Voujeaucourt, France). After freeze‐drying, scaffolds had an average diameter of 9.0 ± 0.8 mm and a thickness of 1.6 ± 0.3 mm (Figure 1a). A central hole (3.5 mm diameter) was created in the scaffolds using a biopsy punch (Dominique Dutscher, Bernolsheim, France).

### 2.2 Human primary cells extraction and culture

SHEDs were collected from deciduous teeth of consenting young patients who underwent dental procedures at the APHP‐Université Paris Cité in Paris, France (Ethical agreement n°DC‐2009‐927, Cellule Bioéthique DGRI/A5, Paris, France) as previously described.^30^ Teeth were decontaminated with povidone‐iodine solution (Betadine, Meda Pharma, France); then the pulp tissues were enzymatically digested with type I collagenase (3 mg mL; Worthington Biochem, Freehold, NJ, USA) and dispase (2 U mL; Roche, Mannheim, Germany). SHEDs were expanded in 150 cm^2^ cell culture flasks (Techno Plastic Products AG, TPP, Trasadingen, Switzerland) at a density of 10^4^ cells cm^−2^ at 37°C (95% air, 5% CO_2_) using Dulbecco’s modified eagle medium (DMEM) (low glucose, 1 g L^−1^) (Gibco by Thermo Fisher Scientific France, Asnières‐sur‐Seine, France), supplemented with 10% (mL mL^−1^) fetal bovine serum (FBS) (Dominique Dutscher, Dubernolsheim, France), and 1% (mL mL^−1^) penicillin and streptomycin (10,000 units mL^−1^–10 mg mL^−1^) (PAN‐Biotech GmbH, Aidenbach, Germany).

Human BMSCs were extracted from the surgical waste of consenting patients undergoing surgery in the femoral head at the University Hospital Center of Bordeaux (UHCB), Bordeaux, France. Cells were expanded in 150 and 300 cm^2^ cell culture flasks (Techno Plastic Products AG, TPP, Trasadingen, Switzerland) at a density of 5 × 10^5^ cells cm^−2^ at 37°C (95% air, 5% CO_2_) using minimum essential medium, alpha modification (αMEM) (nucleosides, no ascorbic acid) (Gibco by Thermo Fisher Scientific France, Asnières‐sur‐Seine, France), supplemented with 10% (mL mL^−1^) FBS (Dominique Dutscher, Dubernolsheim, France), and 1% (mL mL^−1^) penicillin and streptomycin (10,000 Units mL^−1^–10 mg mL^−1^) (PAN‐Biotech GmbH, Aidenbach, Germany).

HUVECs were also extracted from patients at the UHCB as described by References,^48^, ^49^ Bordeaux, France. HUVECs were expanded in 150 cm^2^ culture flasks at a density of 10^6^ cells cm^−2^ at 37°C (95% air, 5% CO_2_) using EBM™^−2^ endothelial cell growth basal medium supplemented with an EGM™^−2^ MV Microvascular Endothelial SingleQuots™ kit (LONZA, Basel, Switzerland).

### 2.3 Cell seeding and spheroid formation

Prior to cell seeding, dried scaffolds were sterilized under an ultraviolet (UV) lamp for 80 min total, 40 min on each side (L‐215.G, Bioblock Scientific by ThermoFisher, Asnières‐sur‐Seine, France). For the spatial rearrangement confocal observations, the cell membranes were labeled with red dye PKH‐26 (SHED) or green dye PKH‐67 (HUVECs) (Sigma‐Aldrich, St. Louis, MS, USA) according to the manufacturers' instructions. For all other experimental settings, cell membranes were not labeled prior to co‐culture. The cells were detached with trypsin/EDTA 0.05%/0.02% (PAN‐Biotech GmbH, Aidenbach, Germany), washed with complete medium, counted, and centrifuged. SHEDs and HUVECs, or BMSCs and HUVECs, were mixed at a 1:1 ratio and cultured for 1, 3, 7, 14, 21, and 28 days at 1 M cells (500,000 HUVECs and 500,000 SHEDs or BMSCs) per each scaffold in a co‐culture media mixture of EBM™^−2^ endothelial cell growth basal medium supplemented with an EGM™^−2^ MV Microvascular Endothelial SingleQuots™ kit (LONZA, Basel, Switzerland) and αMEM (nucleosides, no ascorbic acid) (Gibco by Thermo Fisher Scientific France, Asnières‐sur‐Seine, France), supplemented with 10% (mL mL^−1^) FBS (Dominique Dutscher, Dubernolsheim, France), and 1% (mL mL^−1^) penicillin and streptomycin (10,000 Units mL^−1^–10 mg mL^−1^) (PAN‐Biotech GmbH, Aidenbach, Germany).

The seeding protocol required the suspension of 1 M cells in a volume of 40 μL of co‐culture media mixture for every scaffold. The cells were seeded on top of the surface of the porous scaffolds in 4 directions (10 μL up, 10 μL down, 10 μL right, and 10 μL left). The swelling of the scaffold following the seeding cells, as well as the lack of any adhesion sites, allowed the cells to assemble and aggregate together inside of the pores, compacting into hundreds of spheroids due to surface tension and cohesive forces, in a similar fashion to well suspension methods. The total swelling was obtained by adding 40 μL of medium per scaffold at two different stages following a 10‐min incubation period at 37°C (95% air, 5% CO_2_). For the 3D static culture, seeded porous scaffolds were incubated under standard cell culture conditions in 24‐well plates, 1 scaffold per well, with 2 mL of culture media refreshed every 7 days.

### 2.4 Dynamic culture in a perfusion bioreactor

At 24 h post‐seeding, 21 seeded porous scaffolds were vertically stacked inside a polypropylene (PP) tube (12 mm inner diameter and 4 cm height) and held between two Teflon grids (5 mm height) containing 100 holes of 1.2 mm in diameter.

The flow of culture medium was enabled through the annular space between the inner wall of the PP tube and the edge of the scaffold, along with the inner circular channel created by stacking discs with central holes (Figure 1b). The PP tube was then placed into the bioreactor, which comprised a cylindrical polycarbonate (PC) chamber connected to an external culture medium reservoir via flexible silicone tubes (inner diameter 3.2 mm, R6504‐26BPT, PharMed, Saint‐Gobain Tuyaux, Pont‐à‐Mousson, France). The reservoir contained 100 mL of co‐culture medium, which was refreshed every 7 days, equilibrated at pH = 7.2 with CO_2_‐enriched air (5%) at a 10 mL min^−1^ air flow rate. The silicone tubes were attached to a peristaltic pump (Easy‐load 3, Masterflex, Cole‐Parmer, Chicago, IL, USA) to achieve a perfusion rate of *q* = 10 mL min^−1^. Samples were collected at various time points under sterile conditions by transferring the bioreactor setup under a cell culture hood and using sterile surgical tweezers.

### 2.5 Co‐culture assessment

#### 2.5.1 Cell proliferation assay

Cell proliferation, or total cell number, in different culture conditions was measured using the CyQUANT™ Cell Proliferation Assay (Invitrogen by Thermo Fisher Scientific France, Asnières‐sur‐Seine, France). At every time point, seeded scaffolds were collected from each group then digested using an enzymatic solution containing 10% microbial pullulanase and 5% dextranase (Sigma‐Aldrich, Heidelberg, Germany) in PBS. The cells were washed then frozen at −80°C. On the day of the experiment, samples were prepared according to the manufacturer's instructions to generate triplicates in 96‐well plates. The plates were measured in the Biotek Microplate Reader (Agilent Technologies, Santa Clara, CA, USA), with an approximate fluorescence excitation/emission maximum of 480/520 nm. The experimental setting was repeated in every culture condition at least three times for statistical significance.

#### 2.5.2 Cell viability assessment

Cell viability was assessed using a LIVE/DEAD kit (Invitrogen by Thermo Fisher Scientific France, Asnières‐sur‐Seine, France). Seeded scaffolds were washed then incubated in 500 μL of phosphate‐buffered saline solution (PBS) containing calcein acetoxymethyl ester (calcein‐AM) (1 μL 8 mM) and ethidium homodimer‐1 (2 μL 4 μM) for 90 min on a plate shaker at room temperature to optimize solute diffusion within the scaffold and the spheroids before undergoing confocal observations. At least 30 spheroids were confocally observed per scaffold. The experimental setting was repeated in every culture condition at least three times for statistical significance.

#### 2.5.3 Alkaline phosphatase activity assay

ALP activity was measured as an early osteoblastic marker using the colorimetric ALP Assay Kit (Abcam, Paris, France). The kit uses *p*‐nitrophenyl phosphate (pNPP) as a phosphatase substrate, which turns yellow (max = 405 nm) when dephosphorylated by ALP. The seeded scaffolds were first digested using an enzymatic solution containing 10% microbial pullulanase and 5% dextranase (Sigma‐Aldrich, Heidelberg, Germany) in PBS. The collected cells were then washed twice with PBS, then suspended in 500 μL of ALP assay buffer to undergo cell lysis (Precellys Evolution Touch Homogenizer, Bertin Technologies, Montigny‐le‐Bretonneux, France). The standard and sample wells were then processed according to the manufacturer's instructions. After stopping the reaction, the plates were placed in the Multiskan GO microplate reader (Thermo Fisher Scientific France, Asnières‐sur‐Seine, France) at 405 nm. The enzyme activity was standardized by total protein concentration, detected by a bicinchoninic acid (BCA) protein assay kit (Thermo Fisher Scientific France, Asnières‐sur‐Seine, France). The experimental setting was repeated in every culture condition at least three times for statistical significance. Each sample equates to a singular seeded scaffold.

#### 2.5.4 Human osteopontin assay

Osteopontin (OPN) was measured as an intermediate osteoblastic marker using the Human Osteopontin ELISA kit (Abcam ab100618, Paris, France). The seeded scaffolds were first digested using an enzymatic solution containing 10% microbial pullulanase and 5% dextranase (Sigma‐Aldrich, Heidelberg, Germany) in PBS. Standards and samples were added to wells pre‐coated with antibodies specific to human OPN. After incubation, the wells were washed at least four times with a wash solution to remove unbound material before adding a biotinylated OPN detection antibody. The wells were thoroughly washed again, then an HRP‐streptavidin solution was added. Finally, a tetramethylbenzidine (TMB) substrate reagent was added to develop a colorimetric reaction. After stopping the reaction, the plates were placed in the Multiskan GO microplate reader (Thermo Fisher Scientific France, Asnières‐sur‐Seine, France) at 450 nm. The enzyme activity was standardized by total protein concentration, detected by a BCA protein assay kit (Thermo Fisher Scientific France, Asnières‐sur‐Seine, France). The experimental setting was repeated in every culture condition at least three times for statistical significance. Each sample equates to a singular seeded scaffold.

#### 2.5.5 Human osteocalcin assay

Osteocalcin (OCN) was measured as a late osteoblastic marker using the Human Osteocalcin SimpleStep® ELISA kit (Abcam ab270202, Paris, France). Standards and samples were added to wells followed by a cocktail mix of human OCN capture antibody and human OCN detector antibody. After a 60‐min incubation period, the wells were washed at least four times with a wash buffer before adding a TMB development solution. After stopping the reaction, the plates were placed in the Multiskan GO microplate reader (Thermo Fisher Scientific France, Asnières‐sur‐Seine, France) at 450 nm. The enzyme activity was standardized by total protein concentration, detected by a BCA protein assay kit (Thermo Fisher Scientific France, Asnières‐sur‐Seine, France). The experimental setting was repeated in every culture condition at least three times for statistical significance. Each sample equates to a singular seeded scaffold.

#### 2.5.6 Mineralization

##### 2.5.6.1 von Kossa staining

Seeded scaffolds, fixed in 4% paraformaldehyde (PFA), were stained using the Silver Plating Kit acc. to von Kossa (Sigma‐Aldrich, Heidelberg, Germany). Samples were incubated with silver nitrate solution for 30 min under a UV lamp, followed by a 10‐min incubation with sodium thiosulfate solution. Between steps, scaffolds were rinsed under running tap water. Spheroids were isolated with an enzymatic solution containing 10% microbial pullulanase and 5% dextranase (Sigma‐Aldrich, Heidelberg, Germany) in PBS, then observed using an optical microscope (ZEISS, Axioscope 7, Rueil‐Malmaison, France). The experimental setting was repeated in every culture condition at least three times for statistical significance. At least 20 spheroids were observed for every sample.

##### 2.5.6.2 Alizarin red staining

Matrix mineralization was assessed using Alizarin Red Staining Solution (Sigma‐Aldrich, Heidelberg, Germany) to identify calcium deposits. The solution was diluted 1:1 with distilled water and applied to 4% PFA‐fixed samples for 30 min. Scaffolds were rinsed under running tap water for at least 5 min between steps. Spheroids were then isolated, washed, and observed using an optical microscope (ZEISS, Axioscope 7, Rueil‐Malmaison, France). The experimental setting was repeated in every culture condition at least three times for statistical significance. At least 20 spheroids were observed for every sample.

### 2.6 Immunocytochemistry

In all ICC experiments, scaffolds were collected at all relevant culture conditions and time points then fixed in 4% PFA over a period of 3 h, and washed three times in PBS. Spheroids were isolated from scaffolds using an enzymatic solution containing 10% microbial pullulanase and 5% dextranase (Sigma‐Aldrich, Heidelberg, Germany) in PBS. Spheroids were then suspended and permeabilized in 0.1% Triton X‐100 (Sigma‐Aldrich, Heidelberg, Germany) for 48 h on a plate shaker at 4°C. Following permeabilization, spheroids were repeatedly washed over the course of 24 h. Primary antibodies (Table 1) were then diluted in 0.1% Triton PBS solution and added to the spheroids to incubate overnight on a plate shaker at 4°C. Spheroids were washed again repeatedly overnight, then the secondary antibodies (Table 1) were diluted in 0.1% Triton X PBS solution for overnight incubation. Before confocal acquisitions were taken, spheroids were incubated with 4′,6‐diamidino‐2‐phenylindole (DAPI) (diluted 1/1000 in PBS) for 30 min, then washed three times in PBS. The experimental setting was repeated in every culture condition at least three times for statistical significance.

**TABLE 1:** List of primary and secondary antibodies used for immunocytochemistry experiments.

Protein target	Animal origin	Role	Type	Dilution	References
vWF	Rabbit	Angiogenic marker	Primary	1:200	ab6994, Abcam, Paris, France
CD31	Mouse	Angiogenic marker	Primary	1:200	ab9498, Abcam, Paris, France
NG2	Rabbit	Pericytic marker	Primary	1:100	ab183929, Abcam, Paris, France
αSMA	Mouse	Pericytic marker	Primary	1:200	a2547, Sigma‐Aldrich, Heidelberg, Germany
Collagen type IV	Rabbit	Extracellular matrix (ECM) marker	Primary	1:100	ab6586, Abcam, Paris, France
Anti‐mouse	Rabbit	Fluo 488	Secondary	1:400	a11059, Thermo Fisher Scientific France, Asnières‐sur‐Seine, France
Anti‐rabbit	Goat	Fluo 647	Secondary	1:400	a21245, Thermo Fisher Scientific France, Asnières‐sur‐Seine, France

### 2.7 Confocal image reconstruction, visualization, and spatial analysis

Z‐stack images were acquired during a 20 min period over 370 μm depth by 200 *XY* slices with 1.25 μm resolution (*XY* is parallel to the scaffold base) (Zeiss LSM 700, Rueil‐Malmaison, France). Acquisitions were analyzed in ImageJ 2.14.0/1.54f to create 2D projections and initial 3D evaluations using 3D Viewer and 3DSuite plugins. 3D reconstructions and volume/diameter measurements were done on Avizo 2019.1.

The organization of the endothelial network within the central region of SHED/HUVEC spheroids was quantified using ImageJ (v. 2.14.0/1.54f). From the acquired z‐stacks, middle optical planes were extracted (axial planes). The total spheroid and central regions of interest (ROIs) were manually delineated, and their areas were validated by measurement. The central ROI was defined as the innermost 40% of the spheroid area, with only ROIs corresponding to 40 ± 2% of the total area being accepted for analysis. The red fluorescence channel corresponding to HUVECs was isolated. Images were pre‐processed by contrast increase and Gaussian blur filtering, followed by intensity thresholding to eliminate weak background signals, isolated cells, and small HUVEC clusters. The remaining segmented structures were considered connected ECs. Within the central ROIs, thresholded areas were quantified and expressed as the central HUVEC coverage.

Colocalization and spatial proximity between confocal channels were quantified with Pearson's correlation coefficient using ImageJ (v. 2.14.0/1.54f). The unthresholded coefficient provided a global measure of correlation across the entire image, while the thresholded coefficient excluded background pixels to more accurately reflect correlation between true signal regions. Values approaching +1 indicated strong colocalization or close proximity, whereas values near −1 indicated mutually exclusive localization. Measurements were carried out on axial plane sections of each spheroid.

### 2.8 Data analysis

Results are expressed as mean ± standard deviation (SD). Statistical analysis was performed using two‐way repeated measures analysis of variance with Tukey post hoc tests in IBM SPSS Statistics 29.0.1.0. Graphs were generated on Graphpad Prism 8.0.2. Statistical significance was defined as *p < 0.05; **p < 0.01; ***p < 0.001; ****p < 0.0001.

## 3 RESULTS

### 3.1 Dynamic culture increases long‐term viability of SHED/HUVEC spheroids

**FIGURE 2:**
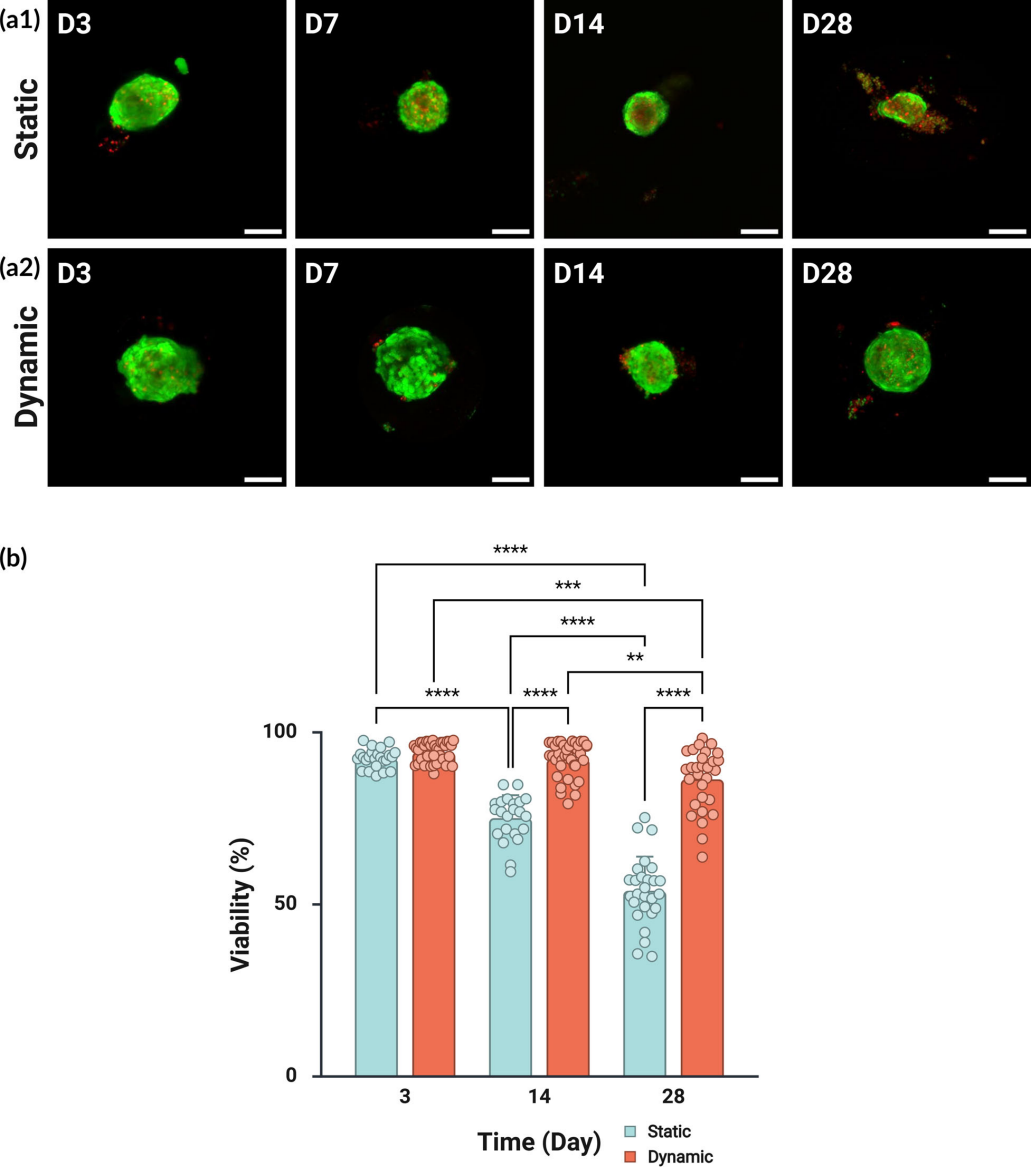
Cell viability. (a) Confocal acquisitions showing the distribution of viable (green) and dead (red) cells using a LIVE/DEAD kit in stem cells from human exfoliated deciduous teeth (SHED)/human umbilical vein endothelial cells (HUVEC) spheroids throughout the culture period under (1) static and (2) dynamic conditions. Scale bar = 100 μm. (b) Quantification of SHED/HUVEC spheroids’ viability (mean ± SD %) under static and dynamic conditions. *n* = 30 spheroids per condition and time point, distributed across three scaffolds (*n*′ = 10 spheroids per scaffold). **, ***, and **** denote *p* < 0.01, *p* < 0.001, and *p* < 0.0001, respectively.

Cell viability was evaluated across different areas of the seeded scaffolds throughout a 28‐day culture period and used to assess the suitability of the 3D culture systems in supporting cell survival. Under static conditions, spheroids quickly formed a dead core after only 1 week of culture (Figure 2a1). The continuous outward spread of dead cells, marked by the red ethidium homodimer‐1 fluorescence, led to their substantial accumulation and to a visible impairment of the geometric integrity of the spheroid by Day 28. Identifying intact spheroids with sufficient compactness for confocal observations was challenging by the end of the culture period in statically cultured scaffolds. Conversely, spheroids cultured under dynamic conditions exhibited dominant green calcein‐AM staining throughout the entire culture period, indicating higher cell viability (Figure 2a2). No necrotic core was observed, with dead cells being randomly distributed across the spheroid.

The red and green channels of the confocal acquisitions were separated to allow quantitative analysis. It is important to note that the viability rates reported throughout the study apply to both SHEDs and HUVECs collectively, as cells were stained indiscriminately for both cell types. According to confocal analysis, cell viability under static culture conditions exhibited a significant and steady decline over time, decreasing from 92.03 ± 3.91% on Day 3 to 53.98 ± 8.79% on Day 28, reflecting a 1.7‐fold total reduction (Figure 2b). In contrast, spheroids cultured under dynamic conditions showed a less pronounced decrease, with loss in viability becoming significant only in the last week of culture. Viability significantly dropped from 95.81 ± 3.68% on Day 3 to 84.71 ± 8.08% on Day 28, a 1.1‐fold total reduction (Figure 2b). Remarkably, spheroids in dynamic groups maintained significantly higher cell viability at every time point compared to those cultured statically.

### 3.2 Dynamic culture prevents cell loss and spheroid fragmentation

**FIGURE 3:**
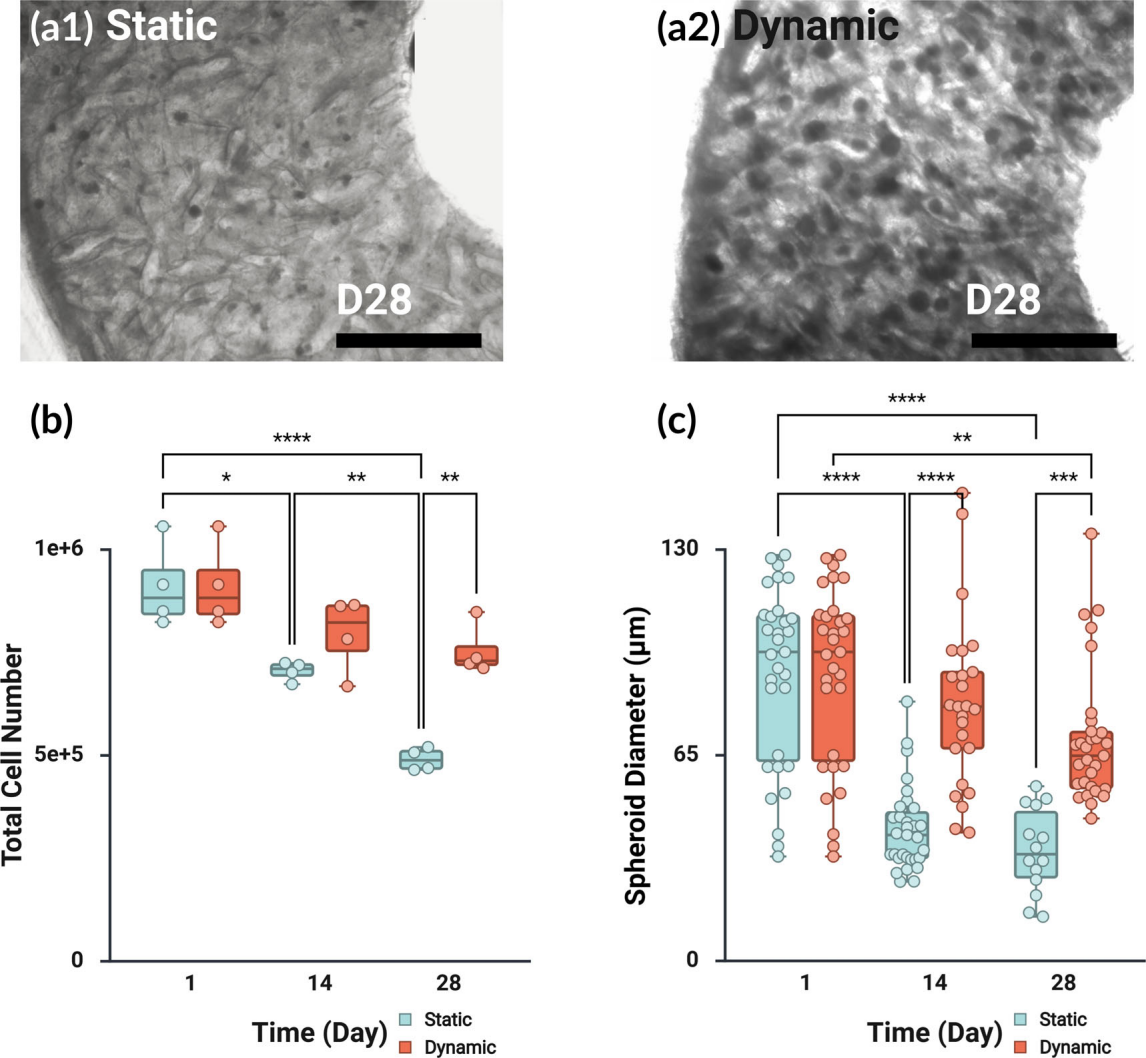
Morphological characterization of the spheroids. (a) White field acquisitions of seeded pullulan/dextran (PUDNA)/hydroxyapatite (HAp) scaffold sections, showing the distribution of stem cells from human exfoliated deciduous teeth (SHED)/human umbilical vein endothelial cells (HUVEC) spheroids under (1) static and (2) dynamic conditions on Day 28. Scale bar = 1000 μm. Evolution of the SHED/HUVEC (b) total cell number per scaffold (mean ± SD cells/scaffold) and (c) spheroid diameter (mean ± SD μm) throughout the culture period under static and dynamic conditions. *n* = 4 scaffolds for cell number assessment, and *n*′ = 30 spheroids distributed across three scaffolds (*n″* = 10 spheroids per scaffold) per condition and time point. *, **, ***, and **** denote *p* < 0.05, *p* < 0.01, *p* < 0.001, and *p* < 0.0001, respectively.

Throughout the culture period, SHED/HUVEC spheroids exhibited significant differences in size and density between static and dynamic conditions (Figure 3a1,a2). Under static conditions, the average cell number per scaffold declined significantly, reaching 709,668.71 ± 20,074.07 cells by Day 14 and further decreasing to 490,471.56 ± 23,744.46 cells by Day 28, corresponding to a 1.9‐fold cell loss from the initial seeding density of 1 M cells/scaffold (Figure 3b). In contrast, under dynamic conditions, cell count per scaffold also declined to 794,804.90 ± 38,322.76 cells by Day 14 albeit not significantly, with 750,145.21 ± 58,966.85 cells remaining per scaffold by Day 28, resulting in a more gradual 1.2‐fold cell loss (Figure 3b). This was further supported by the evolution of spheroid size. Under static conditions, the average spheroid diameter (cubic root of spheroid volume) decreased strongly and significantly from 93.02 ± 22.14 μm on Day 1, to 41.66 ± 13.09 μm on Day 14, and to 36.65 ± 15.47 μm on Day 28, a 2.6‐fold reduction (Figure 3c). In comparison, spheroids cultured under dynamic conditions experienced a significantly smaller reduction in size compared to static conditions (1.3‐fold). Indeed, the average diameter decreased to 84.11 ± 26.90 μm on Day 14, and then significantly to 69.81 ± 21.78 μm on Day 28 (Figure 3c). Moreover, microscopic observations showed that spheroids in the dynamic perfusion group exhibited better compactness, structural integrity, and were higher in number compared to those in the static group.

### 3.3 Dynamic culture favors the development of an inner endothelial distribution

**FIGURE 4:**
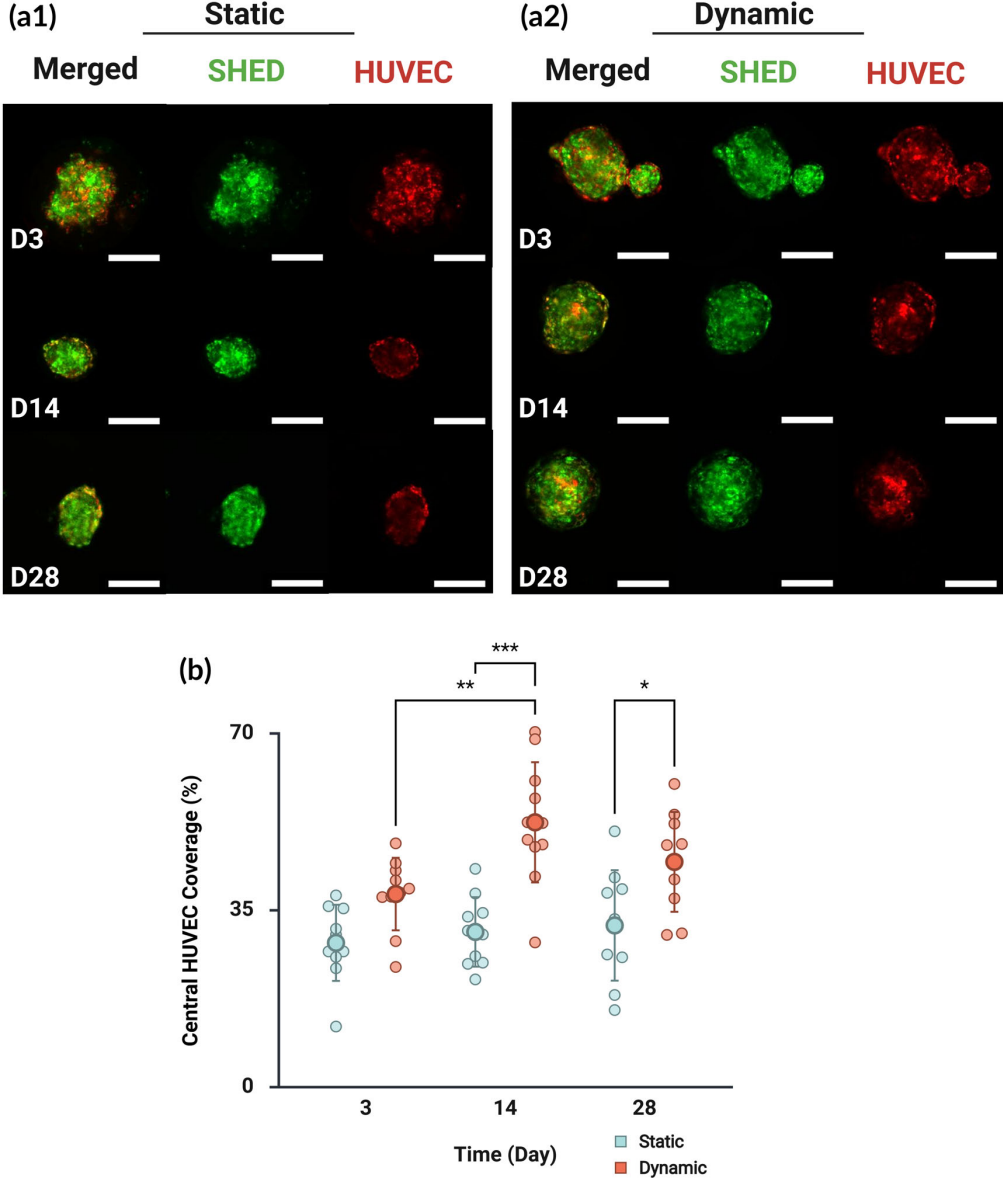
(a) Middle planes of *z*‐stack confocal acquisitions showing the spatial reorganization throughout time of human umbilical vein endothelial cells (HUVECs) stained with PKH‐26 (red) and stem cells from human exfoliated deciduous teeth (SHEDs) stained with PKH‐67 (green) in SHED/HUVEC spheroids under (1) static, and (2) dynamic conditions. Scale bar = 100 μm. *n* = 30 spheroids per condition and time point, distributed across three scaffolds (*n*′ = 10 spheroids per scaffold). (b) Evolution of the endothelial network coverage (mean ± SD %) at the center of SHED/HUVEC spheroids under static and dynamic conditions. *n* = 9 spheroids per condition and time point, distributed across three scaffolds (*n*′ = 3 spheroids per scaffold). *, **, and *** denote *p* < 0.05, *p* < 0.01, and *p* < 0.001, respectively.

Prior to seeding, SHEDs were labeled with green fluorescence (PKH67) and HUVECs with red fluorescence (PKH26) to track their spatial positioning within the spheroids. Confocal imaging at Day 3 revealed a prominent peripheral endothelial envelope formed by HUVECs, enclosing uniformly distributed HUVECs and SHEDs within the spheroid, regardless of culture condition (Figure 4a1,a2). In static culture, this peripheral endothelial arrangement persisted throughout the culture period, with HUVECs failing to invade the spheroid or form dense internal structures (Figure 4a1). In contrast, dynamic culture promoted endothelial redistribution by Day 14, with clusters emerging in the spheroid center (Figure 4a2). By Day 28, this intracentral endothelial network expanded further, becoming denser and more prominent, while SHEDs progressively reorganized toward the periphery.

Quantitative analysis corroborated these observations. Under static conditions, the proportion of the total spheroid area occupied by the endothelial network within the central region remained low and stable, measured at 28.56 ± 7.53% on Day 3, 30.72 ± 6.88% on Day 14, and 32.01 ± 10.93% on Day 28 (Figure 4b). In contrast, dynamic culture induced a marked internal redistribution, with central coverage significantly increasing from 38.22 ± 7.18% on Day 3 to 52.41 ± 11.89% on Day 14. Notably, despite the slight decline in value to 44.59 ± 9.88% on Day 28, values remained significantly higher than those observed in static counterparts at both Day 14 and Day 28, emphasizing perfusion as a driver of network expansion and stabilization.

### 3.4 SHEDs express limited osteogenic markers and fail to achieve mature osteogenic differentiation

#### 3.4.1 ALP expression is weak in both static and dynamic cultures

Following the same experimental settings, BMSC/HUVEC were produced and used as controls to compare osteogenic differentiation. ALP activity was quantified in all co‐cultures under dynamic and static conditions to evaluate osteogenic performance.

**FIGURE 5:**
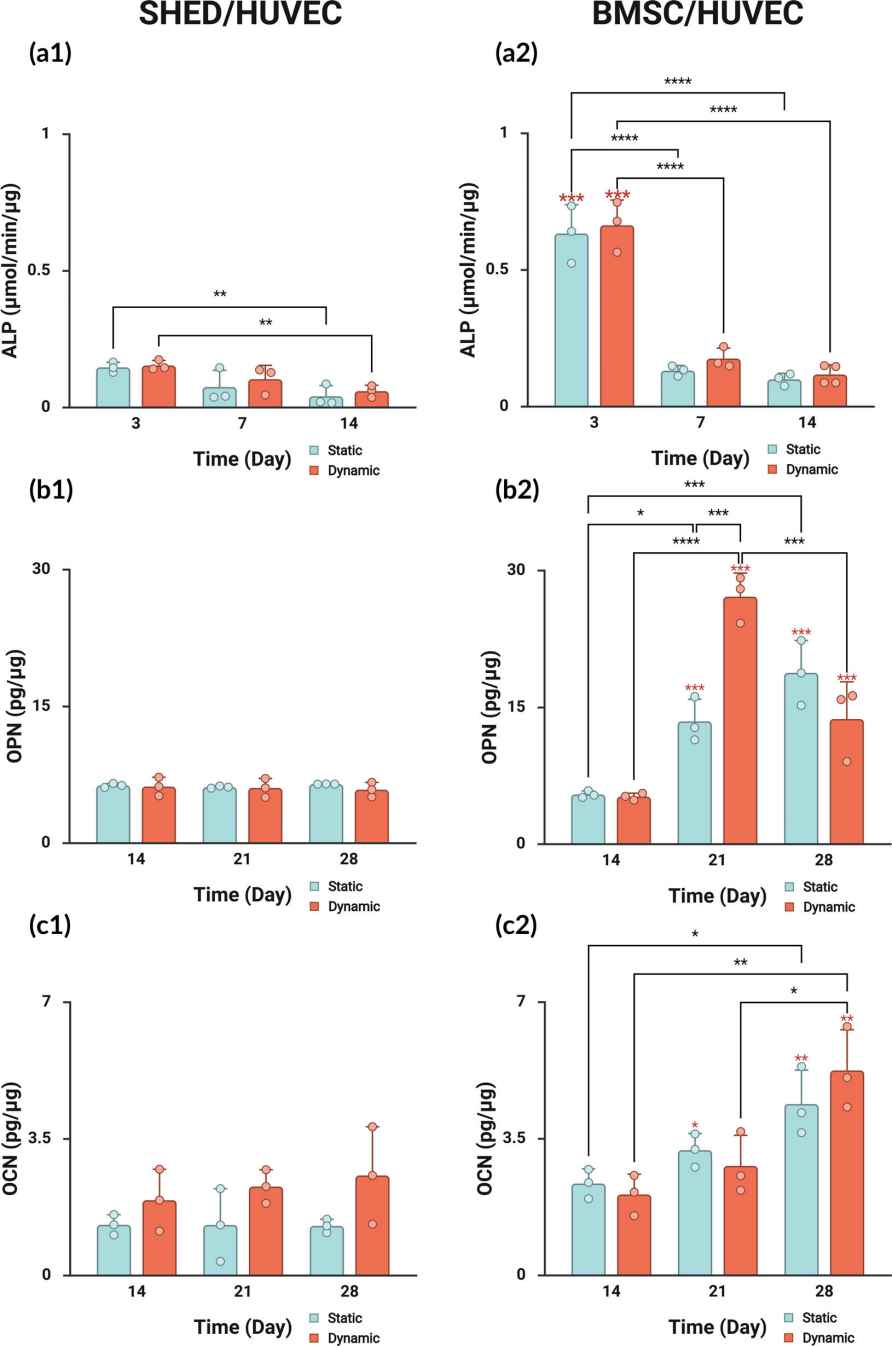
Expression of osteogenic markers during the 28‐day culture period. Standardized (a) alkaline phosphatase (ALP) activity (mean ± SD μmol min^−1^ μg^−1^), (b) osteopontin (OPN) expression (mean ± SD pg μg^−1^), and (c) osteocalcin (OCN) expression (mean ± SD pg μg^−1^) in (1) stem cells from human exfoliated deciduous teeth (SHED)/human umbilical vein endothelial cells (HUVEC) and (2) bone marrow‐derived mesenchymal stem cells (BMSC)/HUVEC co‐cultures under static and dynamic conditions. *n* = 3 scaffolds for each condition and time point. Red asterisks represent statistical comparison between SHED/HUVEC and BMSC/HUVEC co‐cultures. *, **, ***, and **** denote *p* < 0.05, *p* < 0.01, *p* < 0.001, and *p* < 0.0001, respectively.

Under static conditions, ALP expression in SHED/HUVEC spheroids started at 0.15 ± 0.01 μmol min^−1^ μg^−1^ on Day 3, declined to 0.08 ± 0.06 μmol min^−1^ μg^−1^ on Day 7, and then significantly further to 0.04 ± 0.03 μmol min^−1^ μg^−1^ on Day 14 (Figure 5a1). Under dynamic conditions, ALP expression was similarly at its maximum on Day 3 at 0.15 ± 0.01 μmol min^−1^ μg^−1^, before swiftly declining afterwards to 0.10 ± 0.04 μmol min^−1^ μg^−1^ on Day 7, and significantly to 0.06 ± 0.02 μmol min^−1^ μg^−1^ on Day 14 (Figure 5a1).

A similar yet more significant trend was observed in BMSC/HUVEC spheroids (Figure 5a2). Under static conditions, ALP expression reached 0.63 ± 0.08 μmol min^−1^ μg^−1^ on Day 3, before rapidly and significantly declining to 0.14 ± 0.04 μmol min^−1^ μg^−1^ on Day 7, and 0.10 ± 0.02 μmol min^−1^ μg^−1^ on Day 14 (Figure 5a2). Likewise, under dynamic conditions, ALP expression was significantly downregulated from 0.65 ± 0.08 μmol min^−1^ μg^−1^ on Day 3, reaching 0.18 ± 0.02 μmol min^−1^ μg^−1^ on Day 7, and 0.12 ± 0.03 μmol min^−1^ μg^−1^ on Day 14 (Figure 5a2).

No significant differences in early osteogenic ALP expression were observed between static and dynamic conditions, regardless of co‐culture. However, ALP expression was significantly lower in SHED/HUVEC co‐cultures compared to BMSC/HUVEC co‐cultures on Day 3, exhibiting a 4.3‐fold reduction under both static and dynamic conditions.

#### 3.4.2 OPN expression remains minimal even after 28 days of SHED/HUVEC co‐culture

Across both static and dynamic culture conditions, no upregulation of OPN expression was detected in spheroids containing SHEDs, with values remaining close to 5 pg μg^−1^ on Days 14, 21, and 28 (Figure 5b1).

On the other hand, in BMSC/HUVEC co‐culture, OPN expression increased progressively and significantly under static conditions from 5.53 ± 0.29 pg μg^−1^ on Day 14, to 13.48 ± 1.72 pg μg^−1^ on Day 21, and 18.79 ± 3.55 pg μg^−1^ by Day 28 (Figure 5b2). Under dynamic conditions, OPN levels were significantly upregulated from 5.21 ± 0.36 pg μg^−1^ on Day 14, peaking at 27.71 ± 2.08 pg μg^−1^ on Day 21, then declining significantly to 13.79 ± 3.44 pg μg^−1^ on Day 28 (Figure 5b2). Perfusion flow led to an earlier and significantly higher upregulation of OPN expression in BMSC/HUVEC co‐culture compared to static conditions (Figure 5b2).

OPN expression was consistently and significantly weaker in SHED/HUVEC co‐culture compared to BMSC/HUVEC co‐culture during peak activity on Day 21 and Day 28, exhibiting a 3.8‐ and 5.5‐fold reduction under static and dynamic conditions, respectively.

#### 3.4.3 SHEDs' OCN expression in dynamic culture remains significantly inferior to BMSCs

Under static conditions, no upregulation of OCN was observed during the last 2 weeks of SHED/HUVEC co‐culture, with values remaining below 1.3 pg μg^−1^, signifying the baseline (Figure 5c1). Under dynamic conditions, OCN expression showed no significant increase from 1.93 ± 0.79 pg μg^−1^ on Day 14, to 2.31 ± 0.43 pg μg^−1^ on Day 21, and 2.56 ± 1.25 pg μg^−1^ on Day 28 (Figure 5c1).

However, in BMSC/HUVEC co‐culture, OCN levels increased from 2.35 ± 0.37 pg μg^−1^ on Day 14 to 3.11 ± 0.42 pg μg^−1^ on Day 21, and then significantly reached 4.18 ± 0.86 pg μg^−1^ on Day 28 under static conditions (Figure 5c2). OCN expression followed a comparable significantly upward trend under dynamic conditions, increasing from 2.22 ± 0.52 pg μg^−1^ on Day 14, to 2.84 ± 0.78 pg μg^−1^ on Day 21, and 5.54 ± 0.98 pg μg^−1^ on Day 28 (Figure 5c2).

Overall, OCN expression was significantly weaker in SHED/HUVEC co‐culture compared to BMSC/HUVEC co‐culture during the detected period of activation on Day 21 and Day 28, exhibiting a 3.2‐ and 2.2‐fold reduction under static and dynamic conditions, respectively.

#### 3.4.4 No calcium deposits were detected in SHED/HUVEC spheroids

**FIGURE 6:**
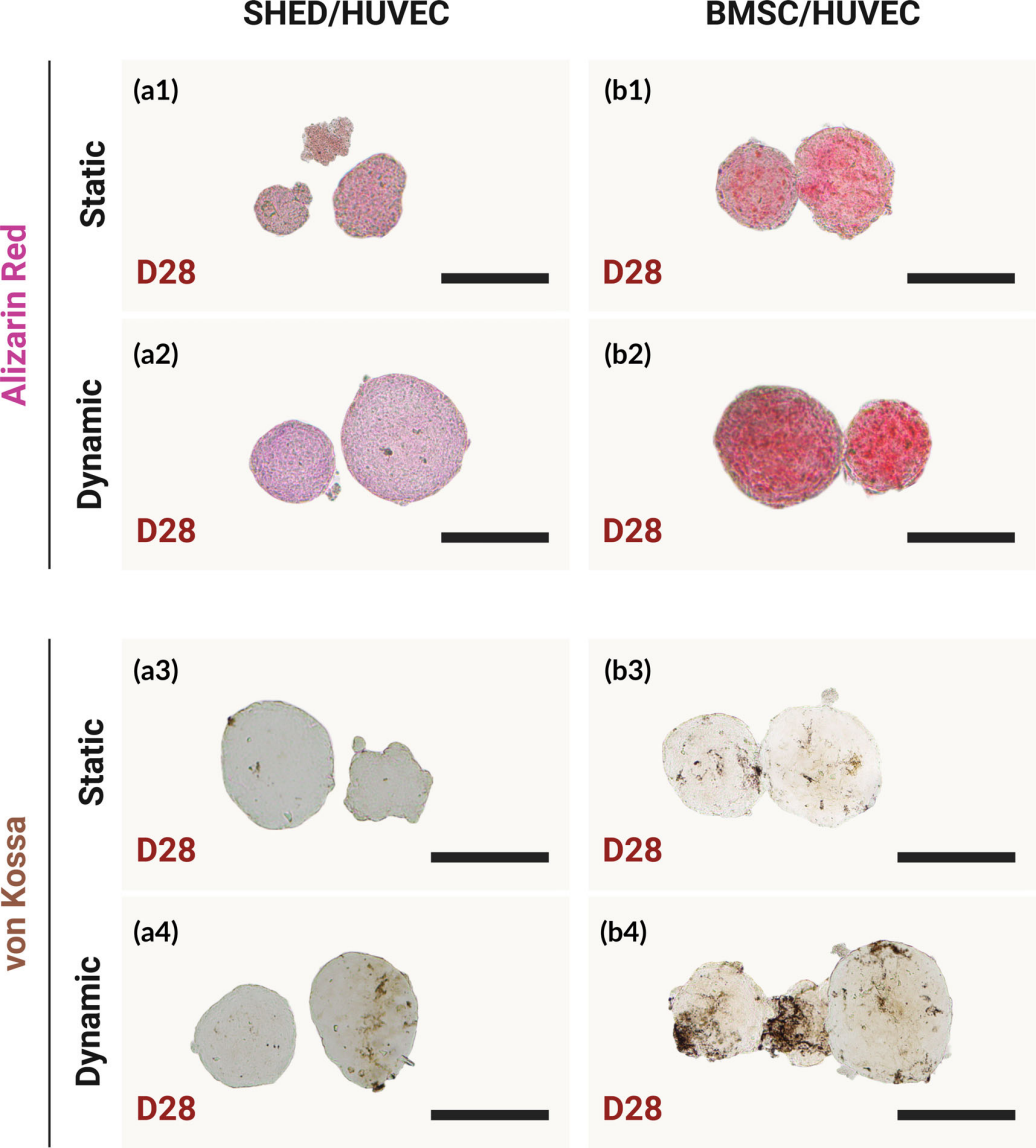
Mineralization assessment. Calcium and phosphate deposits were assessed respectively in (a) stem cells from human exfoliated deciduous teeth (SHED)/human umbilical vein endothelial cells (HUVEC) and (b) bone marrow‐derived mesenchymal stem cells (BMSC)/HUVEC co‐culture spheroids on Day 28 via (1, 2) Alizarin red and (3, 4) von Kossa staining under (1, 3) static and (2, 4) dynamic conditions. *n* = 30 spheroids per condition and time point, distributed across three scaffolds (*n*′ = 10 spheroids per scaffold). Scale bar = 100 μm.

No Alizarin red‐positive red nodules were observed in either static or dynamic groups on day 28, indicating that calcium deposition in SHED/HUVECs spheroids was incomplete or insufficient regardless of culture conditions (Figure 6a1,a2). Comparably, no black/brown phosphate deposits were detected in the static group by Day 28 (Figure 6a3). The dynamic perfusion group exhibited sporadic von Kossa‐positive stains, primarily concentrated toward the center of the spheroids (Figure 6a4). However, these stains were not uniformly present in all spheroids, showing variability in mineral deposition.

Mineralization was also evaluated in BMSC/HUVEC spheroids at the end of the culture period. Abundant red calcium nodules were uniformly distributed throughout the spheroids under both static and dynamic conditions, with a particularly higher density observed in the dynamic group (Figure 6b1,b2). Dark phosphate deposits were also observed in all culture groups, with the largest and most intensely von Kossa‐positive regions appearing in the dynamic BMSC/HUVEC spheroids (Figure 6b3,b4).

To assess the contribution of ECs, SHED monocultures were also evaluated under static conditions. SHED‐only spheroids displayed evenly distributed Alizarin Red‐positive nodules by Day 21, even when maintained in the co‐culture medium, indicating ECM mineralization in the absence of HUVECs (Figure S1).

### 3.5 SHEDs express pericyte markers in all regions of the spheroid

**FIGURE 7:**
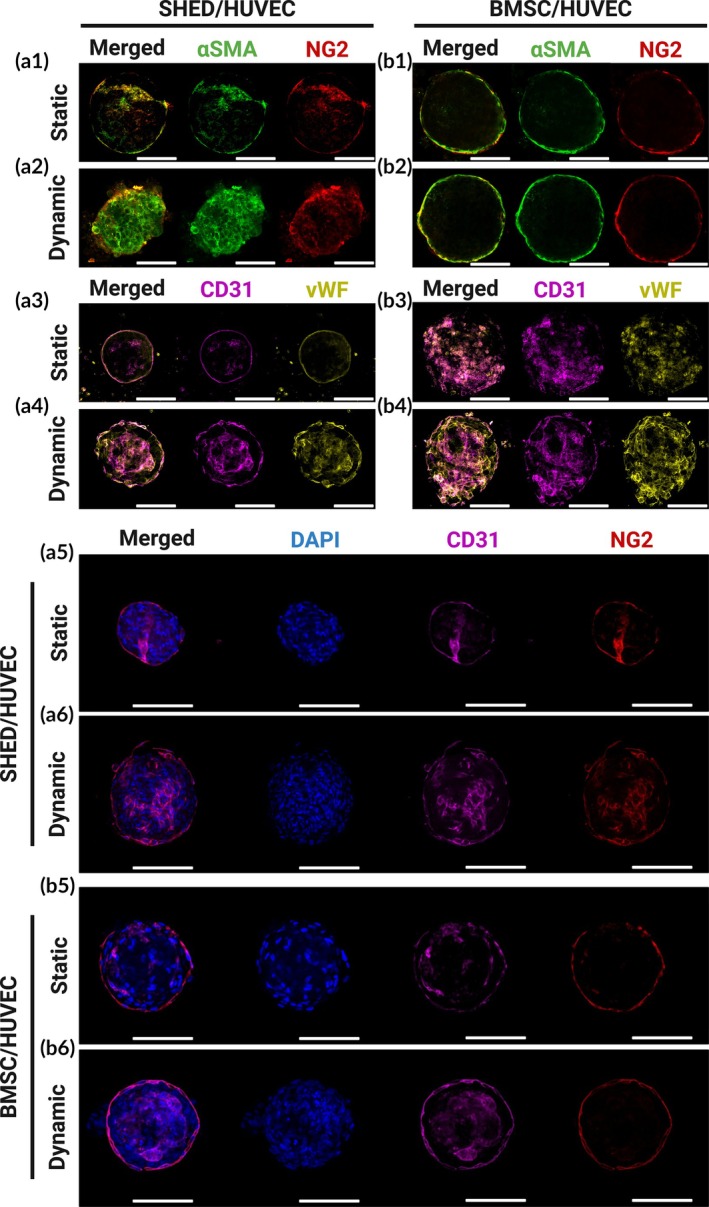
Middle planes from z‐stack confocal acquisitions of (a) stem cells from human exfoliated deciduous teeth (SHED)/human umbilical vein endothelial cells (HUVEC) (b) and bone marrow‐derived mesenchymal stem cells (BMSC)/HUVEC spheroids on Day 28, under (1, 3, 5) static and (2, 4, 6) dynamic culture conditions. (1, 2) Immunostaining of alpha‐smooth muscle actin (αSMA) (green), neural‐glial‐antigen 2 (NG2) (red). (3, 4) Immunostaining of cluster of differentiation 31 (CD31) (magenta), von Willebrand factor (vWF) (yellow). (5, 6) Immunostaining of 4′,6‐diamidino‐2‐phenylindole (DAPI) (blue), CD31 (magenta), NG2 (red). *n* = 9 spheroids per condition and time point, distributed across three scaffolds (*n*′ = 3 spheroids per scaffold) were observed for every immunostaining protocol. Scale bar = 100 μm.

In SHED/HUVEC co‐culture, confocal analysis revealed the expression of neural‐glial‐antigen 2 (NG2) and alpha‐smooth muscle actin (αSMA) throughout the spheroids, suggestive of a potential pericytic phenotype of SHEDs within this co‐culture model. Under static conditions, both markers were detected in the peripheral and central regions, with a strong degree of proximity or colocalization (Figure 7a1). Dynamic conditions further enhanced this distribution, leading to more uniform NG2 and αSMA expression across the entire spheroid, with a higher proximity between the two channels at the periphery (Figure 7a2).

In contrast, BMSC/HUVEC spheroids showed a more restricted expression pattern of these markers. NG2 and αSMA were exclusively localized to the spheroid periphery, with minimal to no signal found in the central areas (Figure 7b1,b2).

SHED monocultures were also examined under static conditions. In contrast to SHED/HUVEC co‐cultures, no NG2 signal was detected in SHED‐only spheroids even after 21 days of culture (Figure S2).

### 3.6 NG2^+^ SHEDs were found in close proximity to the endothelial network

Analysis of endothelial markers in SHED/HUVEC co‐cultures revealed distinct differences between static and dynamic conditions. Under static conditions, cluster of differentiation 21 (CD31) and von Willebrand factor (vWF) staining was mainly confined to the spheroid periphery (Figure 7a3). In contrast, dynamic culture conditions supported the presence of the CD31^+^/vWF^+^ cells extending inward throughout the spheroid, indicating enhanced endothelial organization (Figure 7a4). Furthermore, dual staining of CD31 and NG2 suggested a possible pericytic association of SHEDs with ECs under both static and dynamic conditions, with widespread proximity across spheroid sections (Figure 7a5,a6). This observation was supported by colocalization analysis. The unthresholded Pearson's coefficient was high, indicating that NG2 and CD31 intensities varied together across the images. After thresholding, the correlation decreased but remained positive, showing that in signal‐positive regions NG2^+^ and CD31^+^ cells were at least partially associated, consistent with recruitment of NG2^+^ cells around endothelial structures (Table S1).

In BMSC/HUVEC spheroids, dynamic conditions promoted a more structured and cohesive endothelial network (Figure 7b3,b4). While CD31 and NG2 were closely localized at the spheroid margins, their association was scarcely observed in the core, suggesting that interactions may be confined to the outer regions (Figure 7b5,b6). Colocalization analysis showed that while the unthresholded correlation was moderately high, applying a threshold yielded negative values, indicating that in regions with true signal, NG2^+^ and CD31^+^ populations were predominantly segregated (Table S1).

To further evaluate the effects of spatial proximity between NG2^+^ and CD31^+^ cells, Col IV distribution was assessed. A Col IV network was detected in the center of SHED/HUVEC spheroids, particularly under dynamic conditions (Figure S3). In contrast, Col IV^+^ regions were absent from BMSC/HUVEC spheroids under either culture condition.

## 4 DISCUSSION

In this study, we investigated the osteogenic and angiogenic potential of SHEDs in a spheroid co‐culture system with HUVECs within a composite PUDNA/HAp hydrogel, using BMSC/HUVEC co‐culture as a positive control. The scaffold, lacking cellular adhesion sites, allowed spontaneous spheroid formation and cellular reorganization according to intrinsic properties, while the incorporation of HAp recapitulated key features of the native bone microenvironment.^39^, ^40^ To strengthen translational relevance, we deliberately avoided exogenous chemical induction cocktails, which may not translate well clinically. Instead, the design aimed to recapitulate early bone healing conditions, where mineral cues (Ca^2+^, PO_4_^3−^) act as natural drivers of osteogenic commitment.^37^ This design also ensures that the scaffold remains osteoinductive in situ by recruiting endogenous stem cells and promoting osteogenesis through its composite structure. Moreover, HAp‐enriched scaffolds have been consistently shown to have clear advantages in terms of in vivo performance compared to other biomaterials.^50^ In line with our previous study on BMSC/HUVEC spheroids, dynamic culture again proved advantageous over static conditions, enhancing osteogenic and vasculogenic activity and sustaining viability.^43^ Dynamic systems such as bioreactors, microfluidics, and agitation platforms improve oxygenation, nutrient delivery, and waste removal, thereby preserving spheroid integrity and preventing necrosis, which maximizes the system's performance in vivo.^51^, ^52^, ^53^ Consistently, dynamic perfusion in the present study fostered a supportive environment for SHED/HUVEC spheroids, minimizing fragmentation and sustaining long‐term viability.

With respect to osteogenic performance, progressive markers (ALP, OPN, and OCN) were assessed at different stages of the culture period and under both static and dynamic conditions. SHED/HUVEC spheroids consistently exhibited lower levels of these markers and showed minimal matrix mineralization, whereas BMSC/HUVEC spheroids displayed robust expression, particularly under dynamic culture. In most in vitro and in vivo studies utilizing SHEDs and DPSCs for bone regeneration, differentiation kits or osteogenic supplements such as ascorbic acid, dexamethasone, and β‐glycerophosphate, are routinely employed to induce differentiation.^15^, ^25^, ^26^, ^54^, ^55^, ^56^, ^57^, ^58^, ^59^ In the absence of strong inductive cues, HAp crystals alone appear insufficient to drive osteogenic commitment in SHED/HUVEC co‐cultures. However, SHED monoculture spheroids within PUDNA/HAp scaffolds showed consistent positive Alizarin Red staining, indicating successful mineralization and osteogenic differentiation, outperforming their co‐culture counterpart. This suggests that SHEDs, while retaining osteogenic capacity in isolation, may preferentially adopt alternative roles in the presence of ECs. This behavior can possibly be related to the perivascular origin of SHEDs.^60^ Indeed, DPSCs and SHEDs co‐cultured with HUVECs have demonstrated functional similarity to pericytes, exhibiting a comparable molecular profile and exerting similar chemotactic effects on HUVECs to promote angiogenesis.^28^, ^60^

During angiogenesis, a myriad of angiogenic cues stimulate pericyte proliferation and recruitment to the nascent vascular walls, a critical step for stabilizing and maturing new blood vessels.^61^, ^62^ To assess the pericytic behavior of stem cells co‐cultured with HUVEC, we analyzed the expression of classical mural cell markers NG2 and αSMA in SHED/HUVEC and BMSC/HUVEC spheroids. Confocal imaging showed widespread expression of NG2 and αSMA in SHED/HUVEC spheroids compared to BMSC/HUVEC spheroids, with distribution further enhanced under dynamic conditions. The pericytic profile of NG2^+^ SHEDs was further evidenced by their close spatial proximity with CD31^+^ HUVECs, suggesting a potential perivascular recruitment. SHED monocultures, by comparison, displayed little to no NG2 expression. These findings indicate that the presence of HUVECs drives SHEDs toward a pericytic behavior supporting endothelial organization and intercellular connections. Consistently, Col IV networks were detected exclusively in SHED/HUVEC spheroids, particularly under dynamic conditions, indicating progressive ECM maturation and the transition of ECs toward a vascular profile.^63^ These results are in line with several mechanistic observations previously reported in the literature. SHEDs have been shown to secrete higher levels of MCP‐1 and extracellular domain of sialic acid‐binding immunoglobulin‐type lectin 9 (ED‐Siglec‐9) compared to BMSCs,^64^ which are molecules that respectively act as direct mediators of angiogenesis and modulators of VEGF signaling.^65^, ^66^ Comparative studies have also reported higher expression of endothelial‐related markers such as CD31, VEGF receptor 2 (VEGFR2), and vWF in SHEDs than in BMSCs,^67^ as well as increased levels of basic fibroblast growth factor gene expression,^68^ which further supports their pro‐angiogenic potential. Taken together, the results show that the presence of ECs actively redirects SHEDs toward a perivascular‐like affinity, rather than indicating a loss of osteogenic potential. Interestingly, a subpopulation of SHEDs appeared to retain osteogenic differentiation potential under dynamic flow, as evidenced by the presence of von Kossa‐positive regions in SHED/HUVEC spheroids, suggesting that HUVECs do not entirely suppress their osteogenic potential but instead shift the balance of SHEDs' fate. Such context‐dependent behavior has been linked to spatial positioning, with SHEDs and DPSCs located near capillaries adopting pericytic features, whereas those positioned farther away shift toward osteogenic/odontogenic lineages.^28^, ^69^, ^70^

The spatial arrangement of SHEDs and HUVECs was analyzed to better understand how cell positioning also reflects the pericellular environment. In 3D constructs, spatial distribution governs key parameters such as cell–cell signaling, spheroid compactness, nutrient and oxygen diffusion, and ECM deposition, all of which influence overall spheroid functionality. For angiogenesis in particular, ECs must align into interconnected networks to initiate and sustain vascular structures.^71^, ^72^ Early confocal imaging showed a distinct rearrangement of HUVECs at the periphery, forming an endothelial envelope that persisted throughout culture under both static and dynamic conditions. In EC/MSC co‐culture models, it is often observed that ECs migrate toward the surface of the spheroid and form clusters on the inside, as seen in our results.^73^ This outward migration pattern can be partly explained by the differential adhesion hypothesis, which attributes cell sorting to differences in cadherin‐mediated adhesion, leading ECs to accumulate at the surface where they maximize homotypic interactions and minimize surface tension.^72^ At more advanced stages of the culture, however, endothelial structures began to extend toward the spheroid center, suggesting improved migration and organization under perfusion likely attributable to improved oxygen and glucose availability. Large spheroids (~900 μm) typically develop necrotic cores that restrict central endothelial development, while smaller spheroids support deeper organization.^74^ Our previous modeling of BMSC/HUVEC spheroids using Lattice Boltzmann simulations confirmed that oxygen levels decreased by up to 98% from the periphery to the center, yet perfusion flow limited hypoxia (<3.8 × 10^−3^ mol m^−3^) to only 0.01%–5.40% of cells, even in spheroids >200 μm.^43^ Moreover, spheroids formed within PUDNA scaffolds consistently conformed to the scaffold's pore geometry, resulting in a uniform distribution of spheroids with an average diameter of 100 μm post‐seeding. Based on this structural regularity, oxygen transport conditions can be extrapolated to SHED/HUVEC spheroids, supporting the notion that perfusion effectively prevents hypoxia, sustains viability, and promotes endothelial organization throughout the construct. This more homogeneous endothelial structuring, with ECs also present within the spheroid core, provides a favorable environment for the pericytic commitment of SHEDs. Indeed, this spatial distribution statistically increases their direct interactions with HUVECs, thereby promoting perivascular recruitment and mural commitment.

Whereas BMSCs maintained stable osteogenic commitment across all conditions, SHEDs exhibited greater plasticity and context‐dependent responsiveness, with a superior ability to support endothelial maturation. Future studies could harness this spatial adaptability using tri‐culture systems combining SHEDs, BMSCs, and HUVECs under dynamic perfusion, thereby optimizing intercellular interactions. Such an approach would simultaneously exploit the pericytic behavior and ectodermal potential of SHEDs together with the strong osteogenic capacity of BMSCs, advancing the development of clinically relevant, prevascularized, and potentially pre‐innervated bone constructs.^75^, ^76^ In this context, spatial localization emerges as a critical determinant of SHED fate, influencing their differentiation toward perivascular, neural, or osteogenic lineages depending on neighboring cells.

## 5 CONCLUSION

This study provides new insight into the lineage preferences of SHEDs and BMSCs in endothelial co‐culture and highlights the role of spatial organization in modulating angiogenesis and MSC differentiation. We achieved spontaneous formation of SHED/HUVEC spheroids in a 1:1 ratio within a composite PUDNA/HAp scaffold, with perfusion flow markedly improving cell viability and endothelial organization compared to static conditions. BMSCs have been shown to maintain strong osteogenic differentiation both in monoculture and in co‐culture with HUVECs, confirming their consistent osteogenicity. In contrast, SHEDs displayed a context‐dependent response; while they retained osteogenic activity in monoculture, their role shifted toward a perivascular one when co‐cultured with HUVECs.

Overall, the choice of MSC source should be carefully considered when designing bone tissue engineering and regenerative strategies, as BMSCs provide reliable osteogenic outcomes, whereas SHEDs offer promising potential for supporting endothelial organization.

## AUTHOR CONTRIBUTIONS

**SEH** contributed to methodology, software, validation, formal analysis, investigation, data curation, visualization, and writing the original draft and editing. **CG** contributed to methodology, validation, formal analysis, writing the original draft and editing, and supervision. **MBN**, **RL**, and **EC** contributed to methodology and data curation. **CC**, **DL**, **JA**, and **HD** contributed to conceptualization, formal analysis, funding acquisition, resources, supervision, and editing of the draft. **BPDS** contributed to methodology, validation, formal analysis, writing the original draft and editing, project administration, and supervision. **BD** contributed to conceptualization, formal analysis, funding acquisition, project administration, resources, supervision, and editing of the draft.

## FUNDING INFORMATION

This research was funded by the French Agence Nationale de la Recherche (ANR), under grant ANR‐21‐CE18‐0010‐01 (project HydrOs).

## CONFLICT OF INTEREST STATEMENT

Didier Letourneur has shares in the SILTISS Company, which holds four patents on these scaffolds for tissue engineering. The remaining authors declare that the research was conducted in the absence of any commercial or financial relationships that could be construed as a potential conflict of interest.

## Supporting information


**Data S1.** Supporting Information.

## Data Availability

The original contributions presented in the study are included in the article; further inquiries can be directed to the corresponding author.
