# Supplementary material for: SHEDs and BMSCs exhibit distinct lineage preferences in HUVECs dynamic spheroid co‐cultures: vascular versus osteogenic commitment
Source: Bioeng Transl Med. 2025 Nov 17;11(3):e70091. doi: 10.1002/btm2.70091 (PMC13247431; doi:10.1002/btm2.70091)
Supplement: Supplementary file 1 — Data S1. Supporting Information. [file BTM2-11-e70091-s001.docx]

**Supplementary material:**


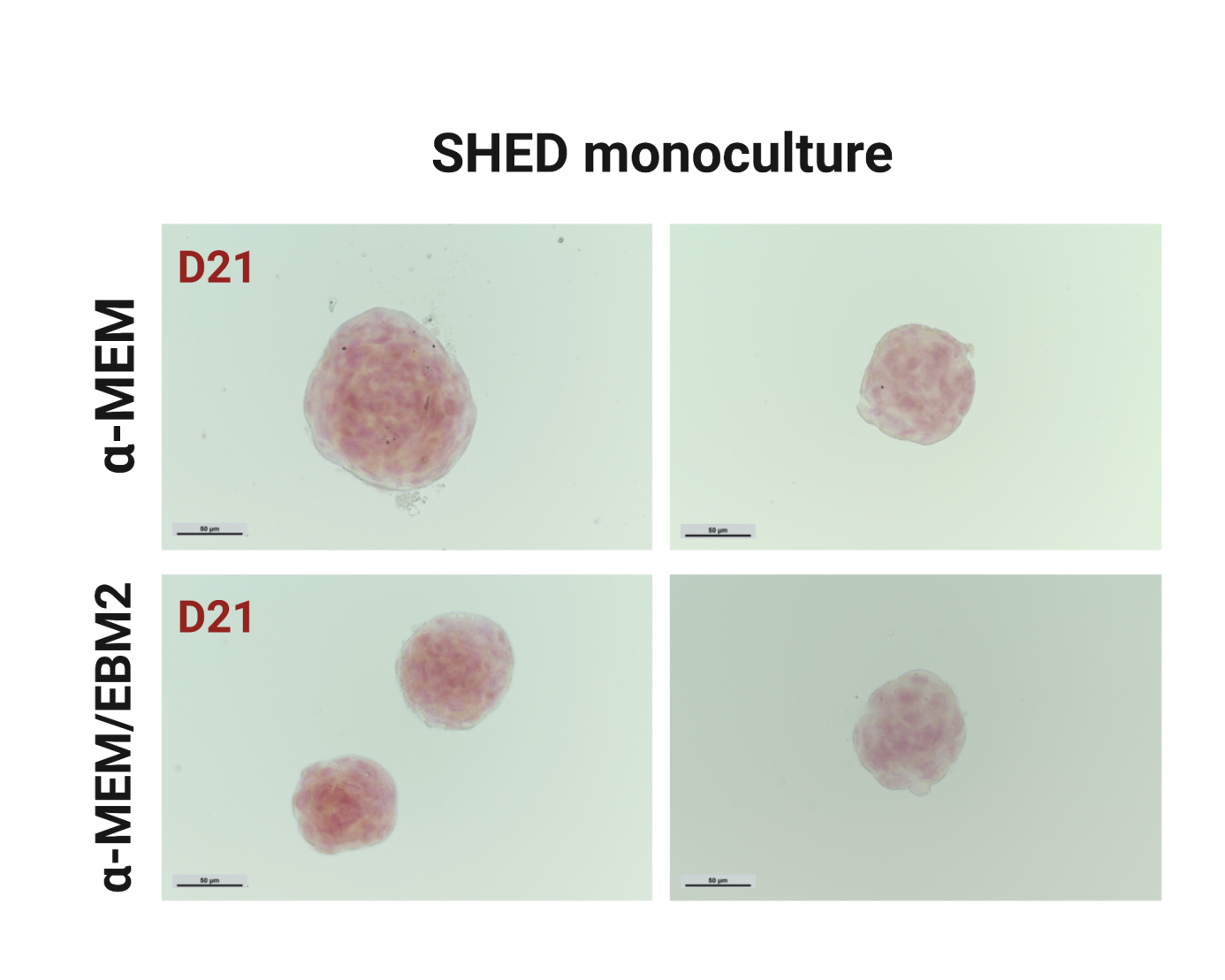


**Figure S1:** Mineralization assessment. Calcium deposits were assessed in stem cells from human exfoliated deciduous teeth (SHED) monoculture spheroids on day 21 via Alizarin red under static conditions using complete minimum essential medium, alpha modification (αMEM), or ɑMEM with EBM™^−2^ endothelial cell growth basal medium (αMEM/EBM2). n = 15 spheroids per condition and time point, distributed across 3 scaffolds (n’ = 5 spheroids per scaffold) were observed for every condition. Scale bar = 50 µm.


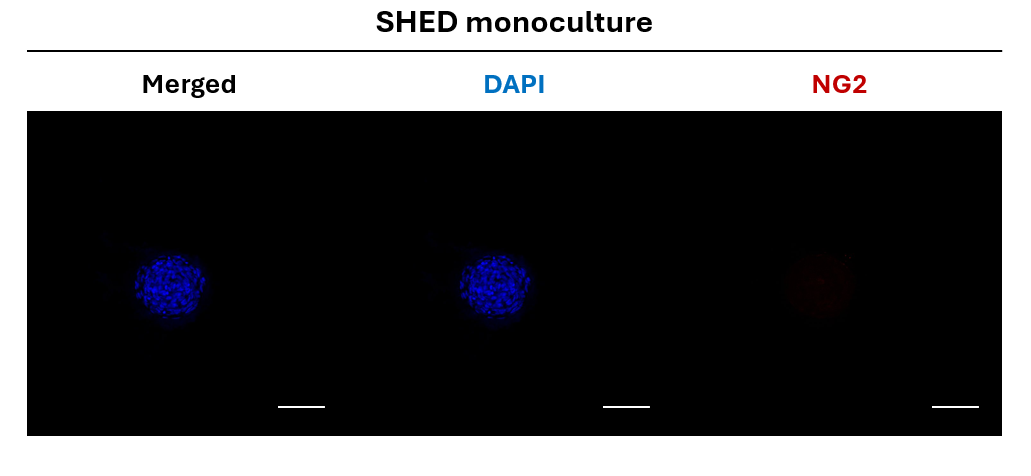


**Figure S2:** Middle planes from z-stack confocal acquisitions of SHED monoculture spheroids on day 21, under static conditions. Immunostaining of 4′,6-diamidino-2-phenylindole (DAPI) (blue) and neural-glial-antigen 2 (NG2) (red). n = 9 spheroids per condition and time point, distributed across 3 scaffolds (n’ = 3 spheroids per scaffold). Scale bar = 100 µm.


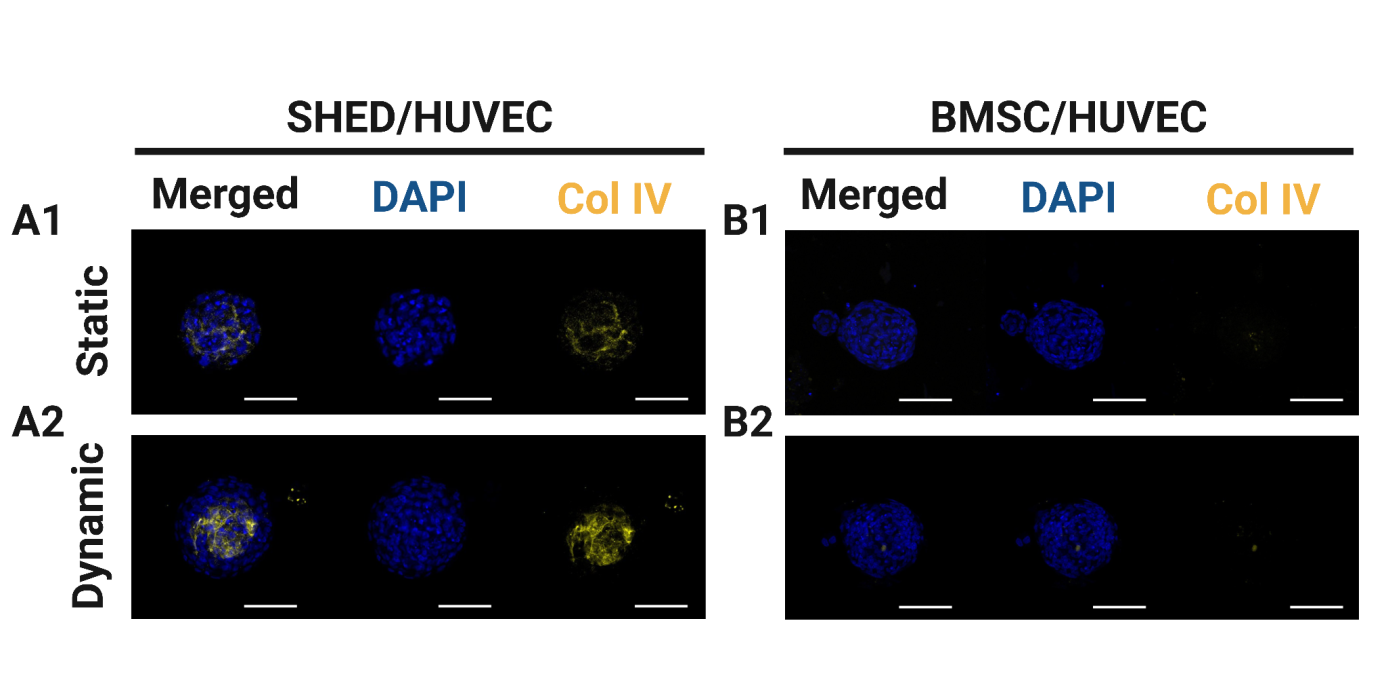


**Figure S3:** Middle planes from z-stack confocal acquisitions of (**A**) stem cells from human exfoliated teeth (SHED)/human umbilical vein endothelial cells (HUVEC) (**B**) and bone marrow-derived mesenchymal stem cells (BMSC)/HUVEC spheroids on day 28, under (**1**) static and (**2**) dynamic culture conditions. (**1**, **2**) Immunostaining of 4′,6-diamidino-2-phenylindole (DAPI) (blue) and collagen type IV (Col IV) (yellow). n = 9 spheroids per condition and time point, distributed across 3 scaffolds (n’ = 3 spheroids per scaffold). Scale bar = 100 µm.

| **Co-culture** | **Condition** | **Pearson R (no threshold)** | **Pearson R (above threshold)** |
| --- | --- | --- | --- |
| SHED/HUVEC | Static | 0.96 ± 0.01 | 0.36 ± 0.07 |
|  | Dynamic | 0.93 ± 0.02 | 0.25 ± 0.08 |
| BMSC/HUVEC | Static | 0.75 ± 0.06 | -0.41 ± 0.18 |
|  | Dynamic | 0.77 ± 0.05 | -0.29 ± 0.06 |

**Table S1:** Pearson correlation of channel 1 (neural-glial-antigen 2 (NG2)) and channel 2 (cluster of differentiation 31 (CD31)) signals in stem cells from human exfoliated teeth (SHED)/human umbilical vein endothelial cells (HUVEC) and bone marrow-derived mesenchymal stem cells (BMSC)/HUVEC spheroids shown in Figure 7(5, 6). n = 9 spheroids per condition and time point, distributed across 3 scaffolds (n’ = 3 spheroids per scaffold).
